# Comparing the extent of breast cancer tumors through contrast-enhanced ultrasound vs B-mode, opposed with pathology: evergreen study

**DOI:** 10.1007/s12282-020-01176-y

**Published:** 2020-10-29

**Authors:** Hiroaki Shima, Toshitaka Okuno, Takashi Nakamura, Aya Noro, Midori Noma, Megumi Sato, Terumi Kaga, Yukio Mituzuka, Keitaro Kamei, Yumi Imayoshi, Toshikazu Ito, Shinsaku Kanazawa, Kumiko Kato, Goro Kutomi, Ryuzo Sekiguchi, Mitsuru Mori, Hasegawa Tadashi, Toshiko Hirai, Ichiro Takemasa

**Affiliations:** 1 Department of Surgery, Surgical Oncology and Science, Sapporo Medical University, S 1, W 16, Chuo-ku, Sapporo, 060-8543 Japan; 2 Breast Surgery, Kobe-City Nishi-Kobe Medical Center, Kobe, Japan; 3 Nabari City Hospital, Nabari, Japan; 4 Mie Prefectural General Medical Center, Yokkaichi, Japan; 5 Hiroshima Prefectural Hospital, Hiroshima, Japan; 6 Hokkaido University Hospital, Sapporo, Japan; 7 Obihiro Kyoukai Hospital, Obihiro, Japan; 8 Toho University Omori Medical Center, Ota-ku, Tokyo, Japan; 9 Ogaki Municipal Hospital, Ogaki, Japan; 10 Rinku General Medical Center, Izumisano, Japan; 11 Breast Surgery, Koga Community Hospital, Yaizu, Japan; 12 Fukui-Ken Saiseikai Hospital, Fukui, Japan; 13 Toho University Ohashi Medical Center, Meguro-ku, Tokyo, Japan; 14 Hokkaido Chitose College of Rehabilitation, Chitose, Japan; 15 Department of Surgical Pathology, Sapporo Medical University, Sapporo, Japan; 16 Department of General Diagnostic Imaging Center, Nara Medical University, Kashihara, Japan

**Keywords:** Breast cancer, Contrast-enhanced ultrasound, Tumor measurement, Pathology

## Abstract

**Background:**

To prove the efficacy of contrast-enhanced ultrasound (CEUS) in determining the extent of resection, more evidence about B-mode and CEUS as opposed to pathology is required. We compared maximum tumor width measured on B-mode/CEUS images with that determined pathologically.

**Methods:**

In this retrospective multicenter study, 152 operable breast cancer patients who had undergone both B-mode and CEUS were analyzed. Maximum tumor width on B-mode and CEUS, and on the postoperative pathological examination (P), was measured by the participating investigators. In addition, maximum width was assessed in B-mode and CEUS image sets by independent reviewers blinded to all patient information. We analyzed differences in maximum width between CEUS, B-mode and P.

**Results:**

The mean widths as measured by the participating investigators were 15 ± 7 mm (B-mode), 19 ± 8 mm (CEUS), and 17 ± 9 mm (P). The difference subtracted P from B-mode was − 3 ± 7 mm (*p* < 0.0001), and that from CEUS was 1 ± 6 mm (*p* = 0.0163). The mean widths as measured by the independent reviewers were 16 ± 7 mm (B-mode) and 18 ± 7 mm (CEUS). The difference subtracted P from B-mode was − 2 ± 8 mm (*p* = 0.0114), while that from CEUS was 1 ± 7 mm (*p* = 0.1921).

**Conclusions:**

Maximum lesion width measurement showed a tendency to increase in the order of B-mode, to P and CEUS. The difference in measurement between P and B-mode was significant, but there was no significant between CEUS and P. These results provide additional information of tendency patterns in measuring the maximum lesion width through enhancement on CEUS.

**Electronic supplementary material:**

The online version of this article (10.1007/s12282-020-01176-y) contains supplementary material, which is available to authorized users.

## Introduction

Contrast-enhanced ultrasound (CEUS) can be obtained using perflubutane as the contrast agent. Perflubutane forms 2–3 µm microbubbles and enables a contrast effect that permits visualization of detailed microvascular perfusion in real time [1–3]. CEUS has recently been utilized in clinical practice for breast cancer screening and treatment, following approval in Japan as evidenced by Phase-2 and -3 studies. Benefits of the technique were expected for mapping the extent of resection, for assessing the effects of systemic treatment [3, 4] and for diagnosis of lymph node metastasis [5, 6]. However, there remains insufficient evidence to confirm its suitability for each of the three potential benefits.

When planning the extent of resection in the case of a partial mastectomy, a comprehensive assessment is performed that includes ultrasound, mammography, and magnetic resonance imaging (MRI). However, each modality utilizes a different imaging process: US originates from ultrasonic agitation, mammography is based on X-ray absorption, and MRI relies on the principle of magnetic resonance. In addition, positional information might differ among these modalities. Therefore, for a realistic comparison, it is necessary to pay close attention to the relative values of tumor size among these diagnostic tools [7, 8]. Pathological findings are derived from the histological evaluation of surgical specimens, which require formalin fixation, histological preparation, and direct visual assessment using a microscope. Thus, a variety of pathological findings are possibly included; however, it is well established that it is very difficult to compare the imaging and pathological findings [9]. Other factors to consider include inherent limitations such as variation in the shrinkage of surgical specimens, and differences between the imaging plane of B-mode/CEUS and that of pathological measurement. For these reasons, the many inconsistencies among the diagnostic modalities remains an unresolved issue.

We focused on comparing between maximal tumor width on B-mode, CEUS and pathological examination (P), as the first step towards achieving a basic data baseline. Therefore, this study sought to compare the lesion widths among them which would lead to the second step of proving the benefit of CEUS in more detail. An exploratory analysis was also attempted to identify the accurate pathological diagnosis and its consistent location information that showed contrast enhancement of CEUS but were not detected on B-mode.

## Patients and methods

We conducted a multicenter retrospective study to compare maximum tumor width among measurements obtained by B-mode, CEUS, and pathological analysis. The patient eligibility criteria were as follows: breast surgery following a pathological diagnosis, complete data regarding tumor width and surgical specimen mapping including documentation and photos, B-mode and CEUS imaging performed prior to breast surgery, and similar probe angles between the B-mode and CEUS images and the direction of pathological excision. We utilized images with relatively aligned directions from three examinations (B-mode/CEUS/pathology) so as to compare widths with similar directions on three planes. The exclusion criteria were prior breast surgery or radiation therapy, and prior systemic therapy in the 3 months preceding surgery. Consecutive breast cancer patients at each institution who met the above criteria were eligible for inclusion in the study. The study protocol was approved by the Clinical Trial Center of Sapporo Medical University, Japan, conducted in accordance with the Declaration of Helsinki and Ethical Principles for Medical Research Involving Human Subjects, and registered with UMIN-CTR (UMIN000028995). The need for informed consent was waived in view of the retrospective and observational nature of the study. An opt-out consent process was used, and disclosures were provided on the University’s website (https://sapmed-surg1.jp/wp-content/uploads/2019/11/282-199.pdf).

All ultrasound systems were equipped with broadband linear phased-array transducers adapted for harmonic imaging. The following systems were used: Aplio 400/500/XG (Canon Medical Systems Corporation, Tochigi, Japan), LOGIQ S8/E9 (GE Healthcare Japan, Tokyo, Japan), HI VISION Preirus (HITACHI, Tokyo, Japan). Contrast-enhanced scanning was performed using amplitude modulation or coded phase inversion harmonic US with mechanical indices of approximately 0.2. At each participatory institution, patients received an intravenous injection of 0.01–0.015 ml/kg of Sonazoid (Daichi-Sankyo Co., Ltd., Tokyo, Japan) prior to the ultrasound examination. Scannings were done in timing as determined individually by the participating investigators, maximum tumor width was measured, and moving and still images were recorded. Key images and case report forms that included each patient’s clinical information and basic clinicopathological factors were collected for analysis from each institution. All identifying information was stripped from the image files used in the evaluation.

**Fig. 1:**
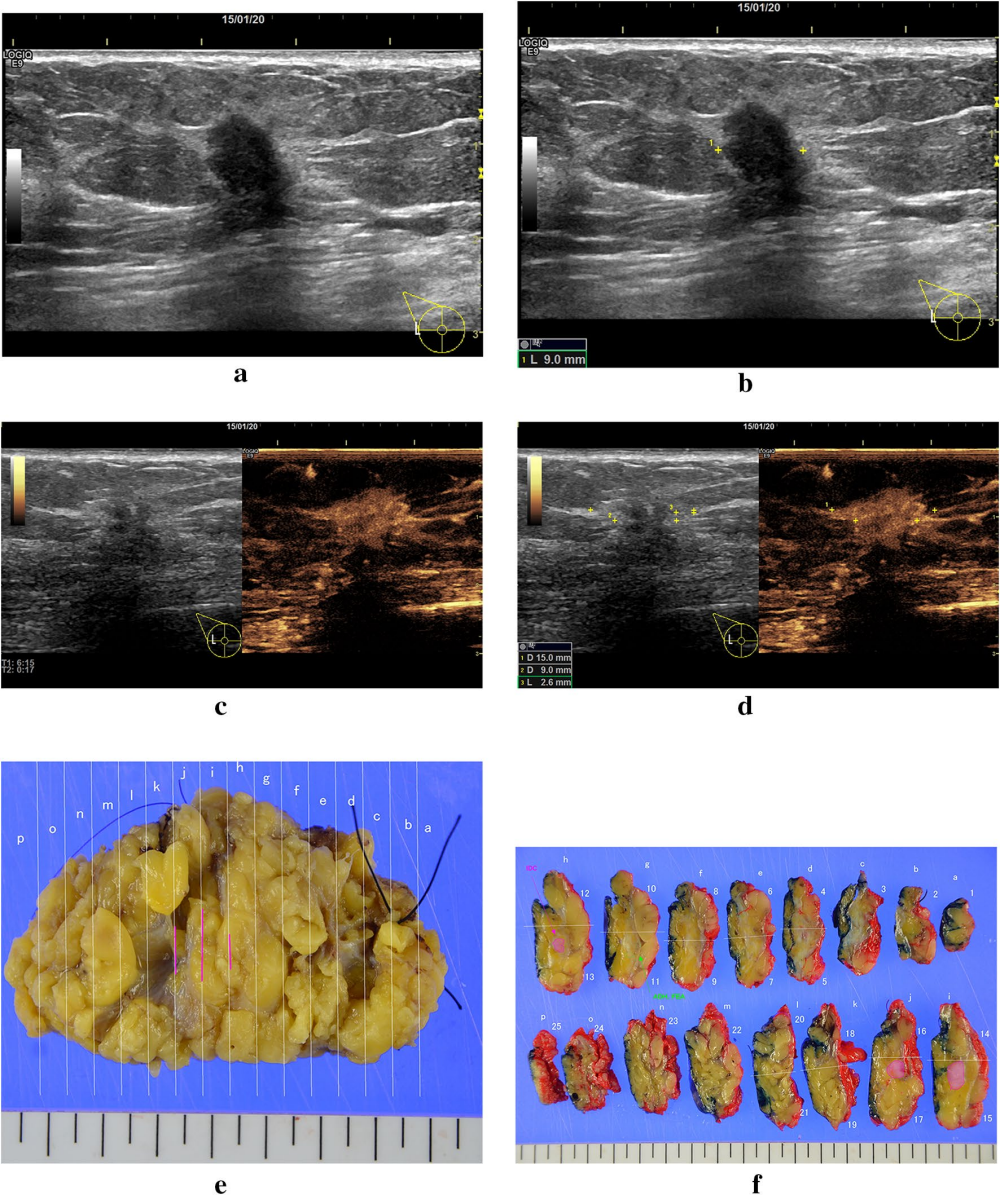
A 59-year-old-woman with right breast cancer had received a partial mastectomy. Her key images described the maximum tumor width was 9, 15 and 11 mm in pre-operative B-mode (**a**, **b**), CEUS (**c**, **d**) and surgical pathological findings (**e**, **f**) measured by one of the participating investigators institutions. Regarding other pathological findings, histology was IDC, ER + , HER2 − and NG1. The maximum tumor width measured by 2 independent reviewer were 9 mm and 16 mm, respectively

Maximum tumor width was obtained from the patients’ pathological reports, photos, and ultrasound images held at the participating institutions (Fig. 1a–f). The following clinicopathological data were collected: age, height, weight, histological findings, estrogen receptor (ER) positivity, human epidermal receptor type 2 (HER2) positivity, nuclear grade (NG), and surgical method. For the exploratory investigation, obvious shrinkage was also reported.

The 153 B-mode and CEUS image data sets were allocated to 5 independent reviewers, who were blinded to all patient information including the clinical and pathological findings. They measured tumor width at its maximum diameter using Image J software (ver. 1.51r, National Institutions of Health, USA [10–12]); the 153 data sets of maximum width were obtained as an average of two width values measured by any 2 out of 5 reviewers. One was excluded because of a size larger than the transducer width. Thus final 152 data sets were then analyzed.

This study aimed to measure the lesion width on B-mode, CEUS and P. Therefore, it was necessary that the evaluated cross sections in the three examinations (B-mode/CEUS/P) should be matched. More precisely, cases that fit the following criteria of the guidelines of Japanese Breast Cancer Society were registered in this study: When partial mastectomy was performed, measurement was made on "perpendicular to the nipple-tumor line", when mastectomy was performed, measurement was made on "direction parallel to the nipple-tumor line".

The primary endpoint was a comparison of maximum tumor width measured by CEUS and that by P (CEUS–P). The secondary endpoint was a comparison of maximum tumor width measured by B-mode and that by P (B-mode–P). We then evaluated whether there was any difference in measured maximum tumor width between CEUS–P and B-mode–P.

### Statistical analysis

All statistical analyses were performed using JMP11 (SAS Institute Inc., Cary NC, USA). The maximum widths of B-mode/CEUS or P are presented as the mean ± standard deviation. The differences between maximal width on B-mode and P, and between maximal width on CEUS and P, were measured and analyzed using a paired *T* test. The correlation between maximum width on B-mode, CEUS, and the pathological findings were analyzed by Pearson product-moment correlation coefficient.

## Results

### Study design

**Fig. 2:**
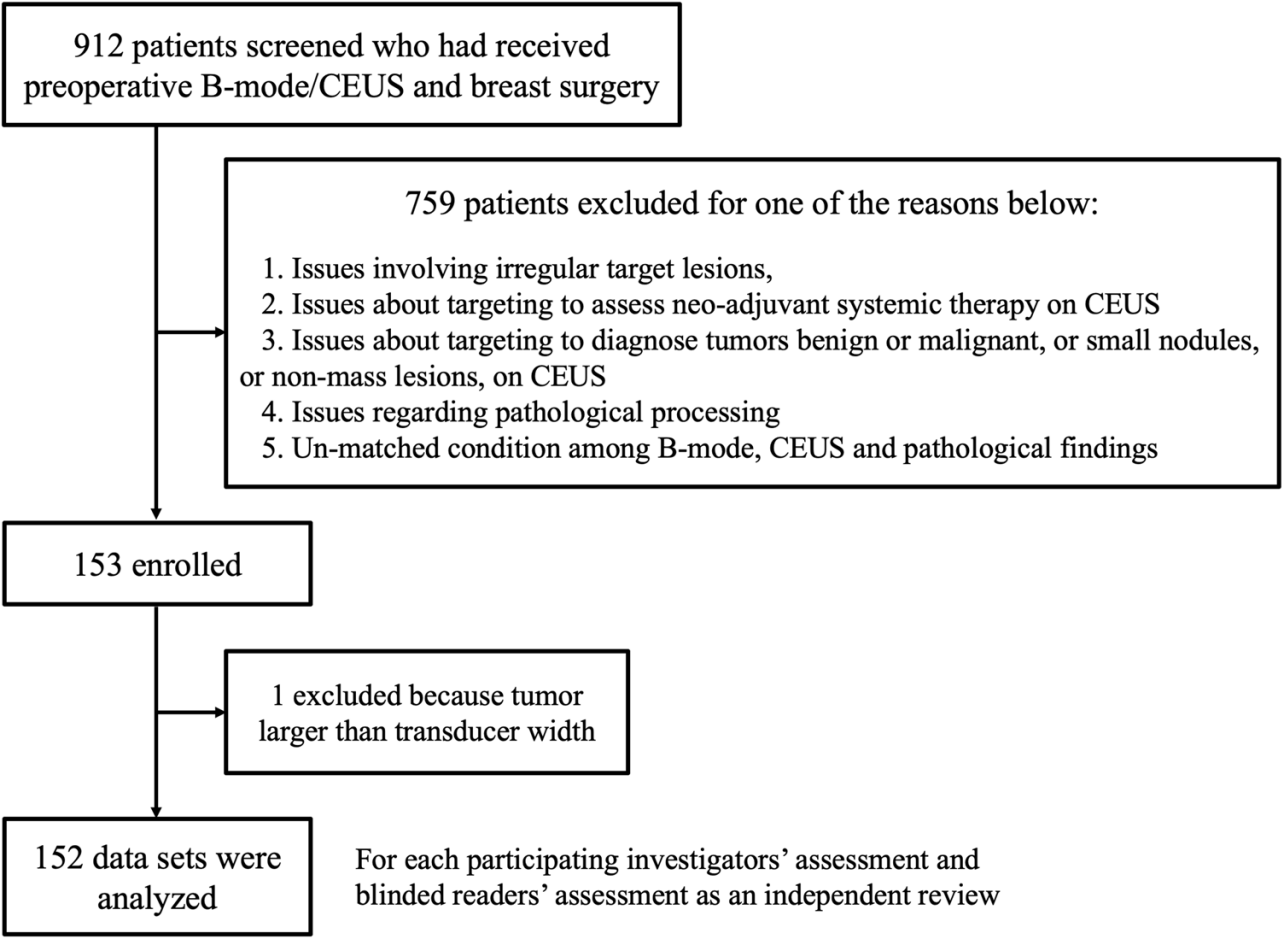
Flow chart of the study design

The median period for registered in each institution was 19 months (1–38 months) between August 2012 and January 2017 at 10 institutions in Japan. 912 patients were screened who had received B-mode/CEUS and breast surgery with primary breast cancer. The most common recruiting issue was the criteria that only those patients be included who had had similar plane B-mode and CEUS, with corresponding pathological findings in the same direction. Accordingly, 759 patients were excluded, and only 153 were enrolled. After excluding one patient in whom the tumor diameter exceeded the width of the transducer, a final total of 152 patient data sets were analyzed. These were measured by participating reviewers and independent reviewers (Fig. 2).

### Patients’ backgrounds

Table 1 lists the patient characteristics. Mean age was 63 years, and mean BMI was 23.4 kg/m^2^. Maximum preoperative tumor diameter on B-mode US was 36 mm. The largest number of diagnoses histologically was invasive ductal carcinoma (*n* = 122), followed by ductal carcinoma in situ (*n* = 11), invasive lobular carcinoma (*n* = 8), carcinoma with apocrine differentiation (*n* = 8) and mucinous carcinoma (*n* = 3). Patients underwent either mastectomy (*n* = 60) or partial mastectomy (*n* = 92).

**Table 1:** Patients’ backgrounds

		*N* = 152
Age	y.o., mean ± SD	63 ± 13
BMI	Kg/m^2^, mean ± SD	23.4 ± 4.0
pT	T1a/T1b/T1c/T2	4/24/73/51
ER	Positive/negative	122/30
HER2	Positive/negative/unknown	26/125/1
NG	1–2/3/unknown	119/24/9
Histology	Ductal carcinoma in situ	11
	Invasive ductal carcinoma	122
	Mucinous carcinoma	3
	Invasive lobular carcinoma	8
	Carcinoma with apocrine differentiation	8
Operation	Mastectomy/partial mastectomy	60/92

*BMI* body mass index, *ER* estrogen receptor, *HER2* human epidermal receptor type 2, *NG* nuclear grade

### Maximum tumor width on B-mode, CEUS, and pathological examinations

**Fig. 3:**
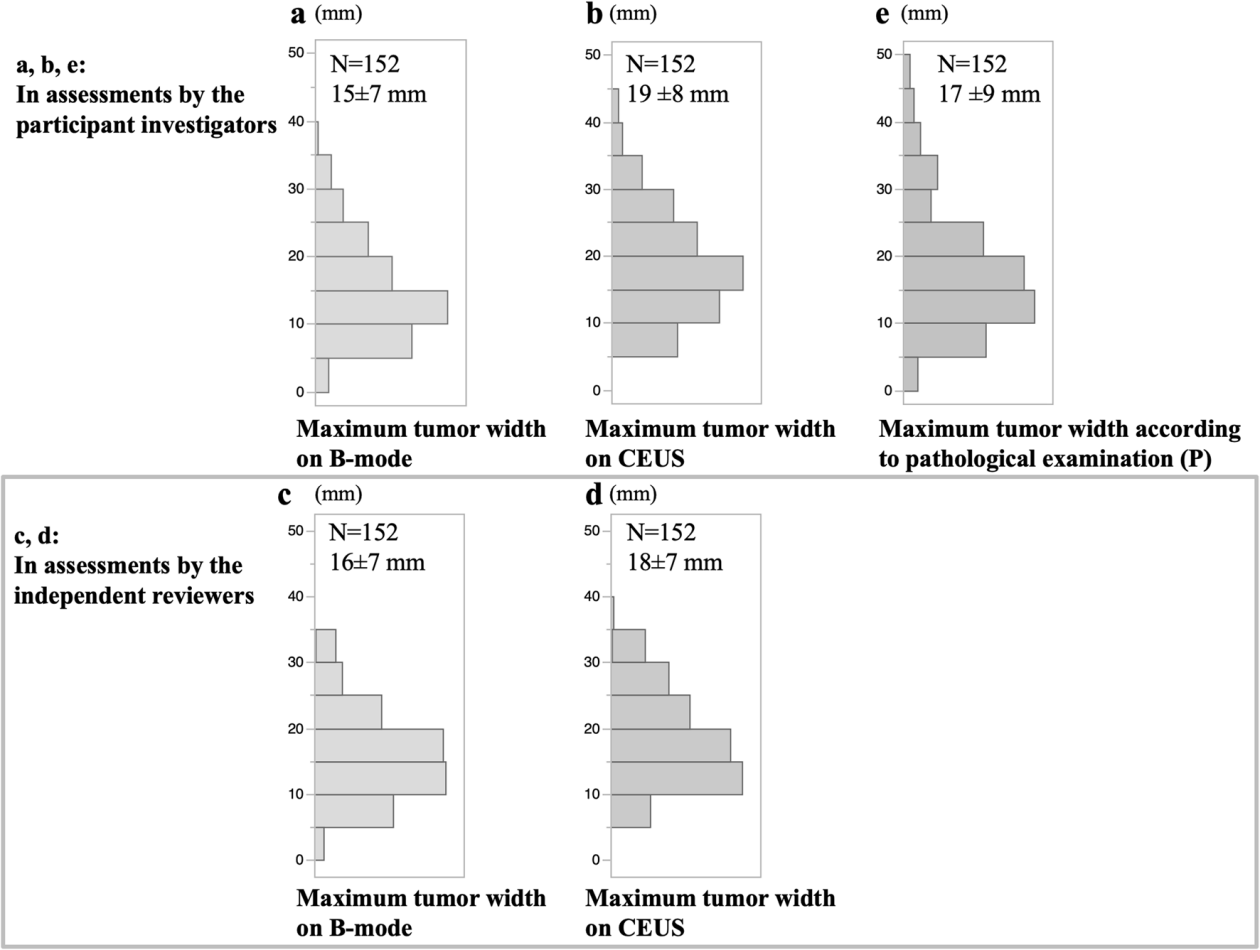
Maximum tumor width on B-mode, CEUS, and pathological examinations. Maximum tumor width on B-mode (**a**) and CEUS (**b**) examinations in assessments by the participating investigators; maximum tumor width on B-mode (**c**) and CEUS (**d**) examinations in assessments by the independent reviewers; maximum tumor width according to the pathological findings (**e**)

The mean tumor widths (± standard deviation) measured by the participating investigators were 15 ± 7 mm (B-mode), 19 ± 8 mm (CEUS), and 17 ± 9 mm (P), as shown in Fig. 3a, b, e. The mean tumor widths measured by the independent reviewers were 16 ± 7 mm (B-mode) and 18 ± 7 mm (CEUS), as shown in Fig. 3c, d. The mean tumor width value was higher on CEUS than on B-mode for both the participating investigators and independent reviewers.

### Difference in maximum tumor width between each of B-mode and CEUS and the pathological findings for a tumor of any size

**Fig. 4:**
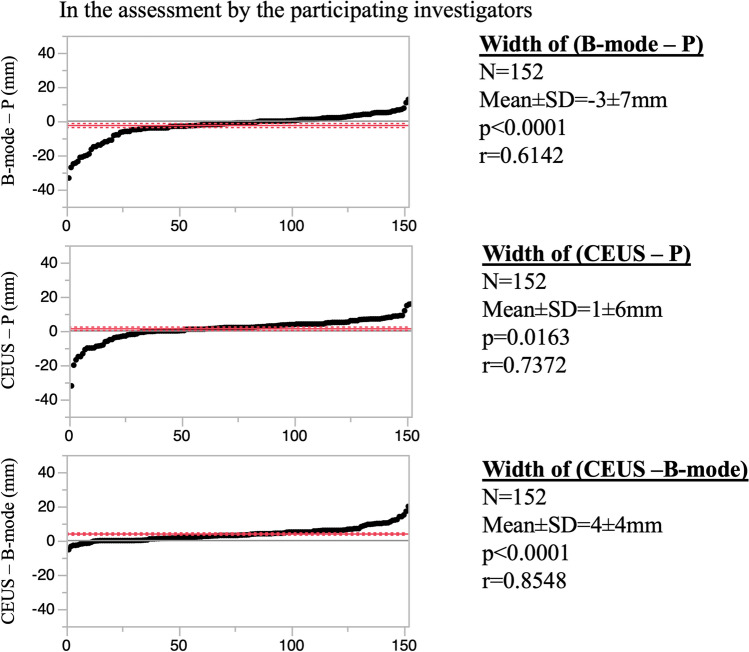
Difference in maximum tumor width between each of B-mode and CEUS and the pathological findings, and between CEUS and B-mode, in the assessments by the participating investigators

**Fig. 5:**
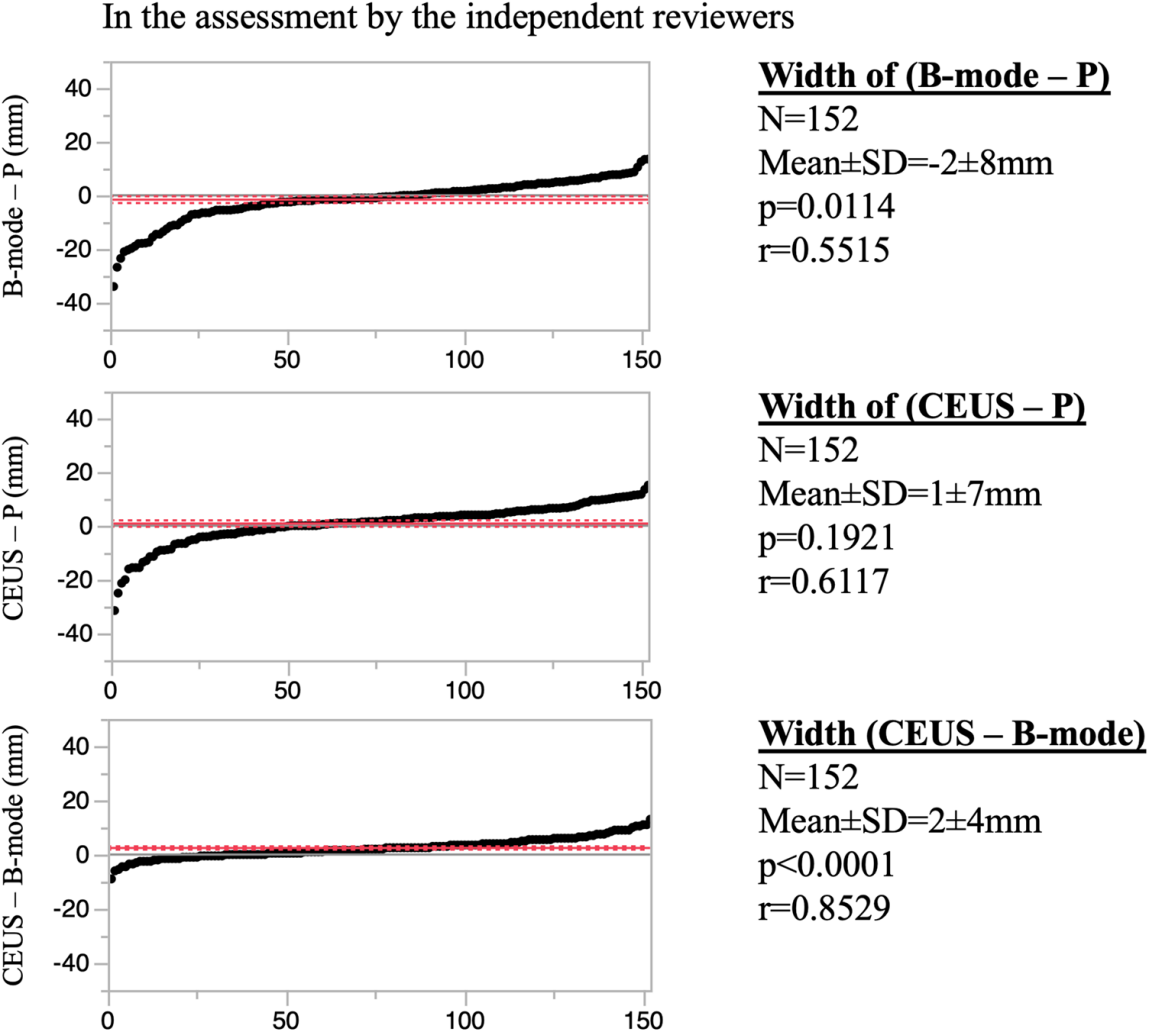
Difference in maximum tumor width between each of B-mode and CEUS and the pathological findings, and between CEUS and B-mode, in the assessments by the independent reviewers

In assessments by the participating investigators, tumor width on B-mode was significantly 3 ± 7 mm shorter than the width of P (*p* < 0.0001), while tumor with on CEUS was significantly 1 ± 6 mm longer than the width of P (*p* = 0.0163). There was a significant difference between the CEUS and B-mode measurements (*p* < 0.0001) (Fig. 4). In assessments by the independent reviewers, the mean difference between the P and the B-mode measurements was − 2 ± 8 mm (*p* = 0.0114) and that between the P and the CEUS measurements was 1 ± 7 mm (*p* = 0.1921). There was a significant difference between the CEUS and B-mode measurements (*p* < 0.0001; Fig. 5). Additionally, there was a correlation among B-mode, CEUS and P with either pattern (Figs. 4, 5).

### Impact of obvious shrinkage on measured tumor width

After excluding 11 data sets that the participating investigators judged to show obvious shrinking of the specimens, 141 of the 153 patient data sets were included in the pathological assessments (Fig. A1). Mean maximum tumor width was 17.7 ± 9.4 mm (Fig. A2b). There was a similar width between this and all included data. Thus, a bias of shrinkage might be of lesser importance, (*n* = 152, Fig. A2a; the same data presented in Fig. 3e).

### Pathological findings in regions on CEUS but invisible on B-mode

In an additional exploratory analysis, we investigated the presence of pathologically cancerous or non-cancerous lesions in areas that showed enhancement on CEUS (invisible in B-mode) but were not visualized outside the area of visible tumor on B-mode. Although 306 regions (right and left regions of the main tumor in the 153 cases) showed enhancement outside the area of visible tumor on CEUS, many were very small in size (mean diameter, 2.8 mm), and only 92 could be diagnosed as cancerous or non-cancerous (Fig. A3). Of these 92 regions, 51 lesions were cancerous (55.4%) and 41 were non-cancerous (44.6%). The pathological findings were as follows: invasive lesions (*n* = 15), in situ lesions (*n* = 12), both invasive and in situ (*n* = 23), and lymphatic invasion (*n* = 1; Table A1).

## Discussion

Although it is of interest to conduct a comparison among the imaging examinations of B-mode/CEUS and the pathological findings, the comparison is difficult when the images and histological sections are in three different planes, particularly in a retrospective analytical setting. The results revealed that maximum tumor width values were highest on CEUS, followed in decreasing order by those on the pathological findings, and B-mode. The widths on B-mode were significantly shorter than the widths measured in pathological findings in both the participating investigators’ assessments and the independent reviews. On the other hand, while the widths on CEUS were statistically 1 mm longer on average than the widths of pathological findings in investigators’ assessment, there was no significant difference between the width on CEUS and P in the independent review. These results suggest that tumor widths according to the pathological cancer lesion might be similar to those on CEUS, but different from those on B-mode. Moreover, there was a positive correlation between the widths on CEUS, B-mode and P with either pattern. Taken together, our results suggest this tendency among the three modalities might be observed in tumors of any size. A previous study reported a tendency for area of enhancement on CEUS to be wider than observed on B-mode, which suggests that the area of enhancement might reflect ductal spread in situ or invasive lesions [13].

In this exploratory study, we focused on areas of enhancement on CEUS that were located outside the tumor circumference that could be visualized on B-mode, and investigated how these areas might correspond with lesions histologically (Fig. A3). These areas of enhancement were pathologically apparent on 92/153 tumors (60.1%), and 55.4% of the enhancing regions were cancerous (Table A1). Possible reasons for the lack of pathological findings in these lesions are that the mean diameter of these areas was 2.8 mm, which is very small compared with the images obtained by B-mode/CEUS and the pathological findings, and also that these small lesions were very difficult to assess retrospectively. Many exclusions occurred because of inapplicable and imponderable circumstances, derived from less possible to identify the pin-point location of these minor diameter differences, and to determine from the pathological diagnosis how the pathological findings correspond to the regions of contrast enhancement in this retrospective setting.

A previous study has also compared maximum tumor diameter among the three different modalities [13]. That study also faced limitations due to inherent issues, and in that sense, the present study is unexceptional. In this study, however, the influences of biases were classified, as shown in Table A2. Two identified biases had only a minor influence on this study. The first issue is shrinkage of the surgical specimen, which might result in loss of landmarks in the pathological examination, and consequently loss the relationship between the images and the pathological findings. Krekel reported minor impact of shrinkage of surgical specimens after formalin fixation [14]. Pritt B. reported that tumors after final processing and mounting decreased in mean width by 2.4 mm compared with the initial fresh measurement in 20 cases (40%), while 21 cases (42%) showed no change, and increased 9 cases (18%) [15]. In our study, maximal mean width excluding 11 data sets with obvious shrinkage (*n* = 141, Fig. A2b) was compared with maximal mean width of original 152 data sets (Fig. A2a), then the difference of tumor width in the pathological findings was ≤ 0.3 mm (Fig. A2). Therefore, shrinkage of the surgical specimens after formalin fixation may have had little effect on the result of the present study. Next, further reduction of bias in the process of assessment was attained, because the tumor widths on the images were measured by participating investigators and independent reviewers.

On the other hand, some biases still remained in this study. First, the very strict inclusion criteria of requiring similar planes among the three modalities in a retrospective setting has obviously caused a selection bias. Second, the most important biases stem from the inclusion of “un-matched condition between three modalities” (Table A2). When comparing tumor sizes between the three modalities; tumor size measurement discrepancies and the corresponding pathologic findings based on the imaging plane, and similar but strictly different planes were observed in B-mode or CEUS despite being in the same position [13]. Third, the regions that showed contrast enhancement of CEUS but were not detected on B-mode, were too small to assess pathologically and resulted in many excluded cases. Thus, these limitations weigh heavily as issues yet to be resolved. Therefore, the basic data itself in these conditional settings may nonetheless be meaningful, and it would allow us to prepare for conducting a prospective study by reference to these retrospective cohorts under certain conditions, implementing comparisons among the three different modalities.

In conclusion, maximum tumor width showed a tendency to increase in the order of B-mode to P and CEUS, and maximum tumor width was similar between CEUS and the pathological findings. We could not conclude superiority of CEUS from only this retrospective study, but our data suggests that a research on enhanced areas provided by CEUS might be worthy of more detailed investigation. Clinical trials are warranted to support the determination of the extent of resection in preparing for partial mastectomies.

## Electronic supplementary material

Below is the link to the electronic supplementary material.

Supplementary Fig. A1: Flow chart of the exploratory study to investigate the effect of surgical specimen shrinkage on measured tumor width (TIFF 13737 kb)

Supplementary Fig. A2: Impact of obvious shrinkage on measured tumor width. Maximum tumor width for all included data (which was the same data presented in Fig. 3e) (a), Maximum tumor width excluding 11 pathological data sets in which obvious shrinkage was observed (b) (TIFF 13737 kb)

Supplementary Fig. A3: Flow chart of the exploratory study to investigate regions visible on CEUS but invisible on B-mode (TIFF 13737 kb)

Supplementary file4 (TIFF 13737 kb)

Supplementary file5 (TIFF 13737 kb)
