# Supplementary figures and images for: Comparing the extent of breast cancer tumors through contrast-enhanced ultrasound vs B-mode, opposed with pathology: evergreen study
Source: Breast Cancer. 2020 Oct 29;28(2):405–13. doi: 10.1007/s12282-020-01176-y (PMC7925467; doi:10.1007/s12282-020-01176-y)

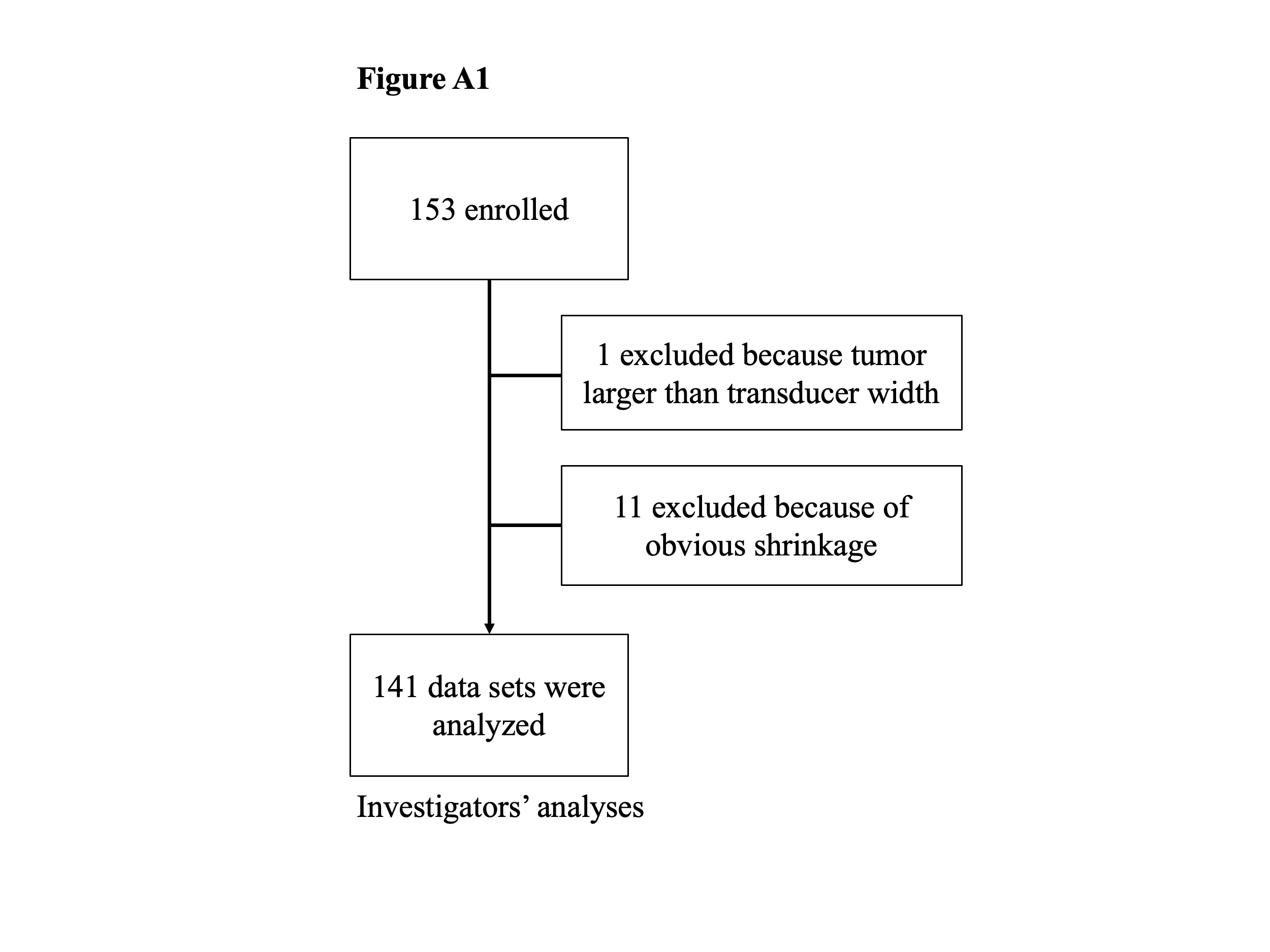

Supplement: Supplementary file 1 — Supplementary Fig. A1: Flow chart of the exploratory study to investigate the effect of surgical specimen shrinkage on measured tumor width (TIFF 13737 kb) [file 12282_2020_1176_MOESM1_ESM.tiff]

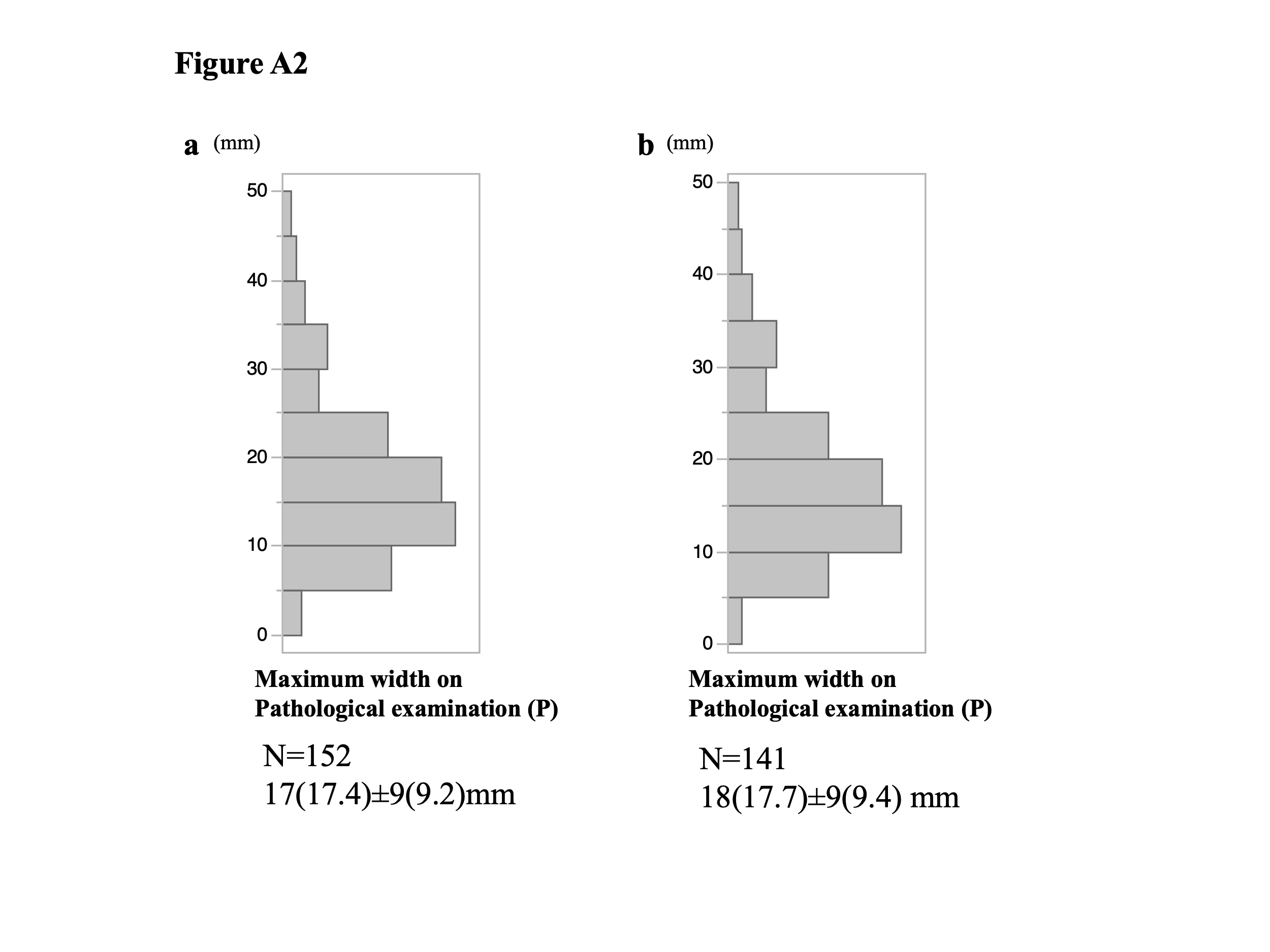

Supplement: Supplementary file 2 — Supplementary Fig. A2: Impact of obvious shrinkage on measured tumor width. Maximum tumor width for all included data (which was the same data presented in Fig. 3e) (a), Maximum tumor width excluding 11 pathological data sets in which obvious shrinkage was observed (b) (TIFF 13737 kb) [file 12282_2020_1176_MOESM2_ESM.tiff]

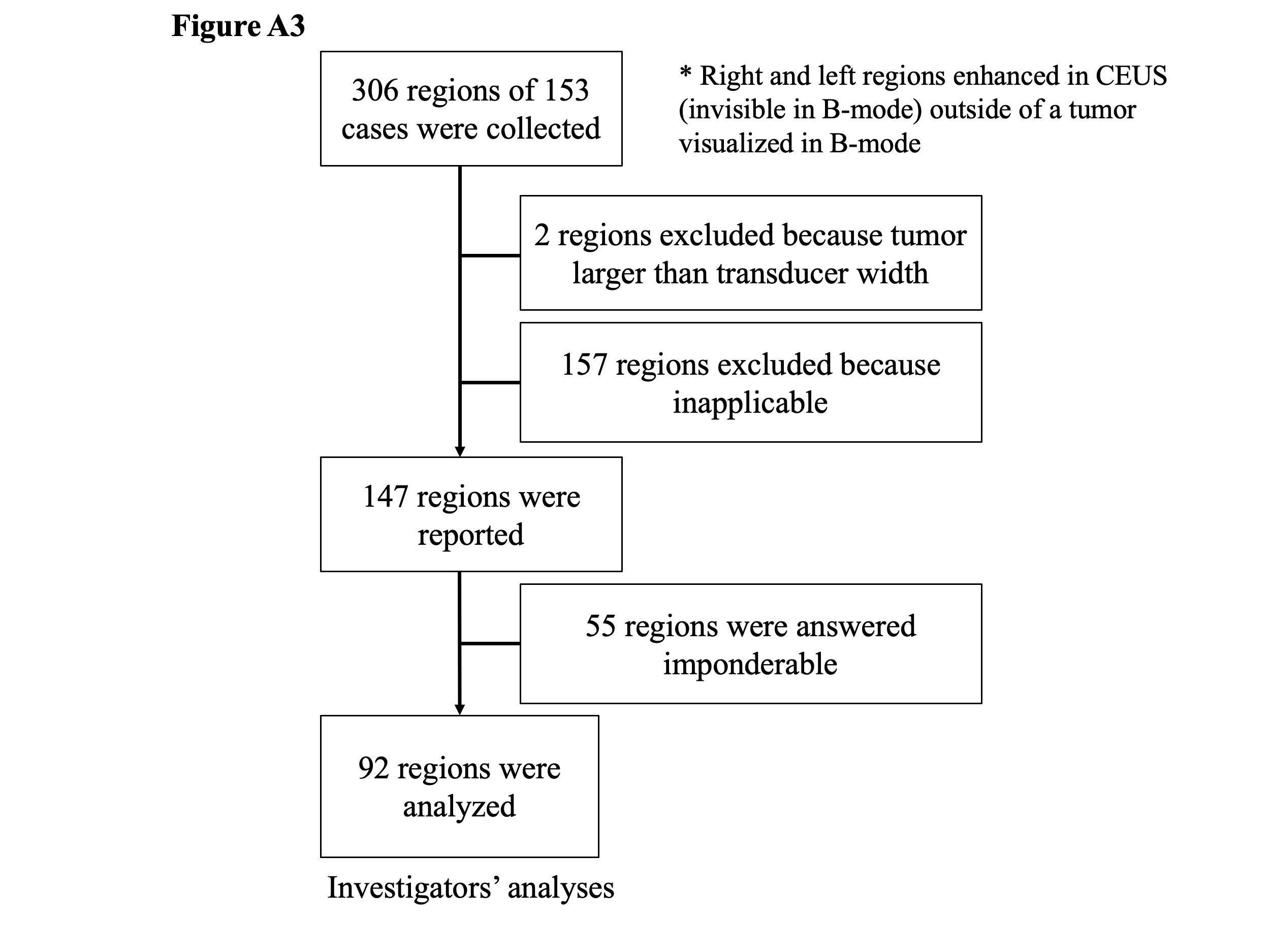

Supplement: Supplementary file 3 — Supplementary Fig. A3: Flow chart of the exploratory study to investigate regions visible on CEUS but invisible on B-mode (TIFF 13737 kb) [file 12282_2020_1176_MOESM3_ESM.tiff]

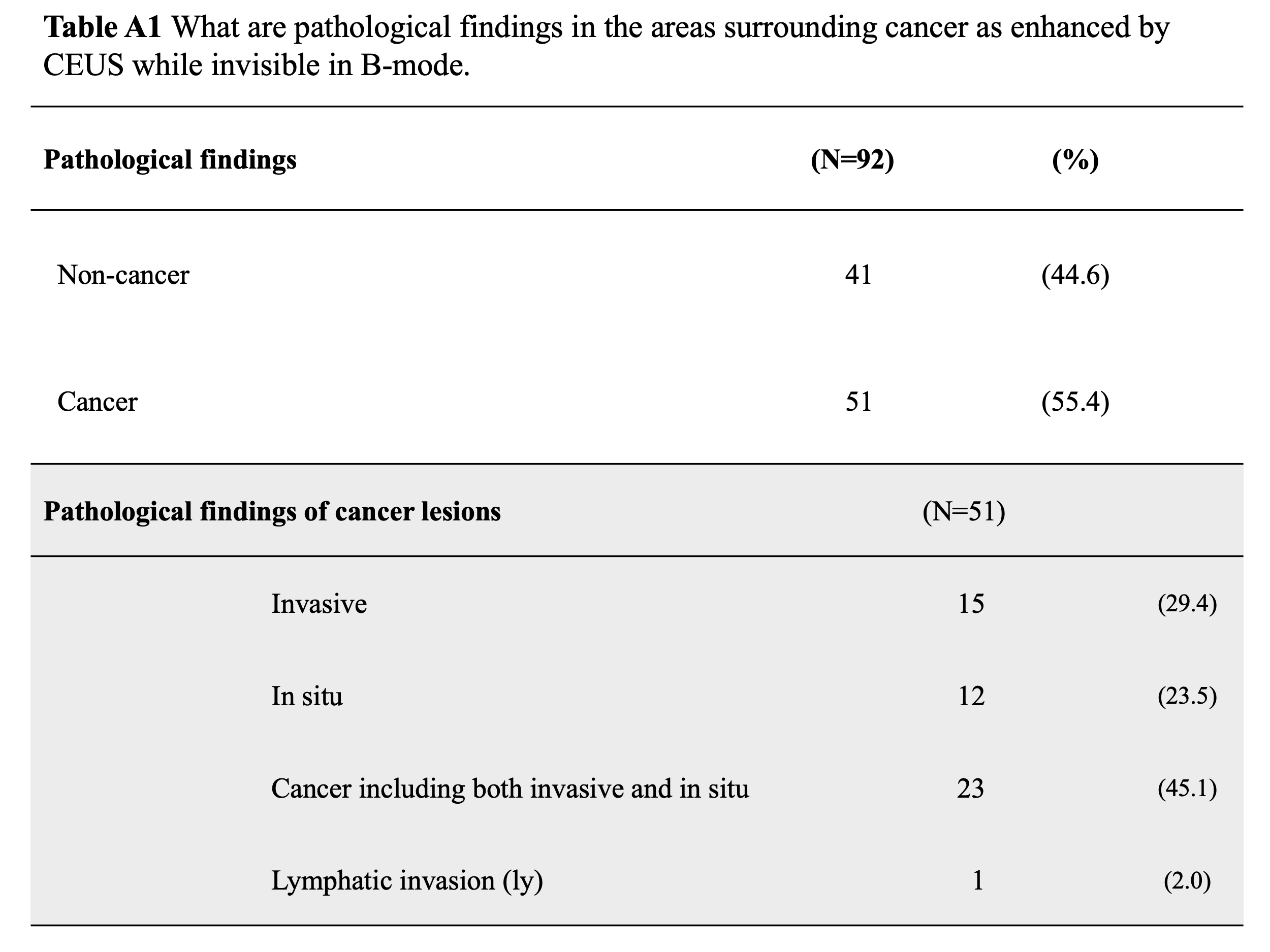

Supplement: Supplementary file 4 — Supplementary file4 (TIFF 13737 kb) [file 12282_2020_1176_MOESM4_ESM.tiff]

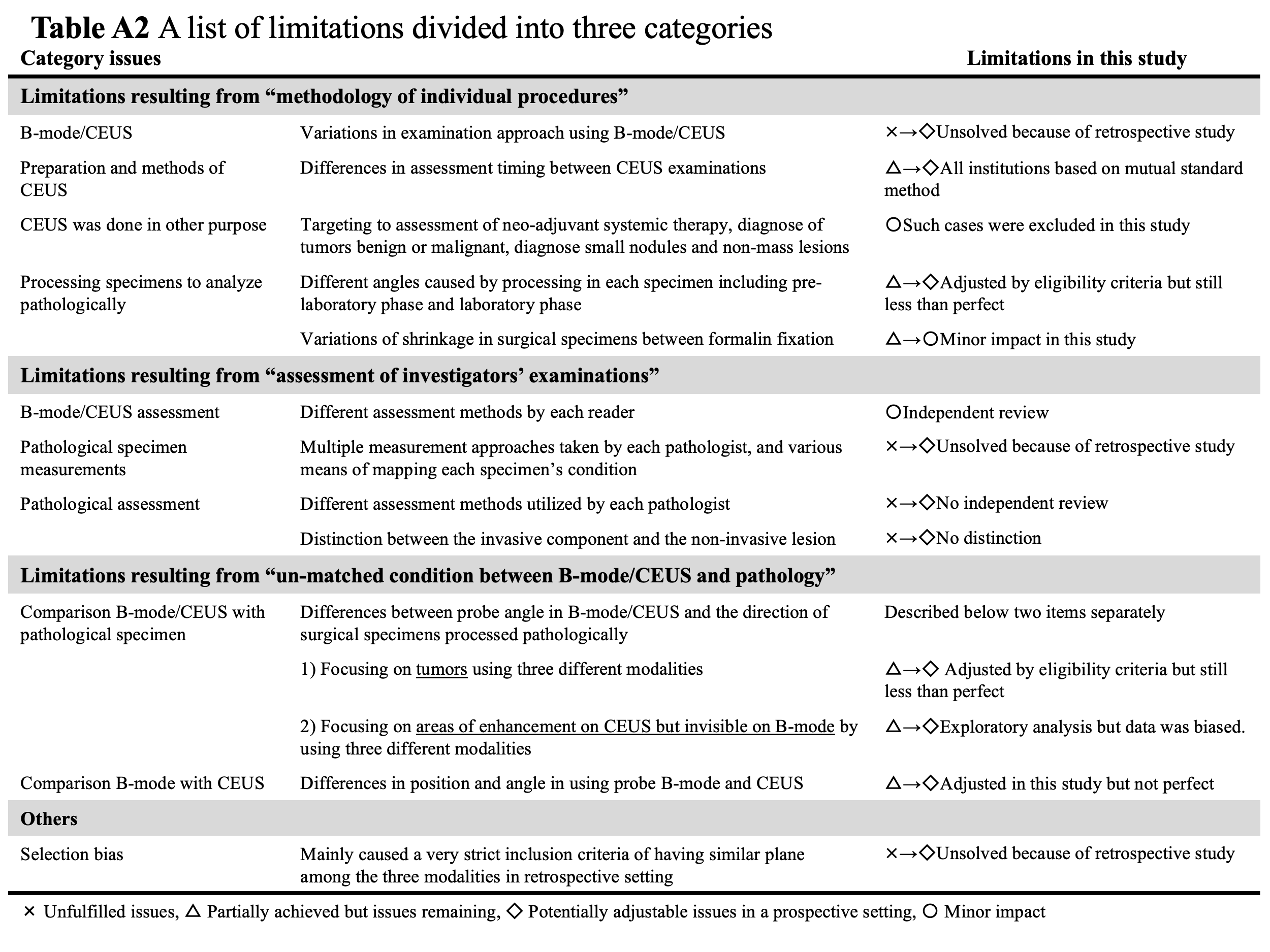

Supplement: Supplementary file 5 — Supplementary file5 (TIFF 13737 kb) [file 12282_2020_1176_MOESM5_ESM.tiff]
